# Assessing sleep consciousness within subjects using a serial awakening paradigm

**DOI:** 10.3389/fpsyg.2013.00542

**Published:** 2013-08-20

**Authors:** Francesca Siclari, Joshua J. LaRocque, Bradley R. Postle, Giulio Tononi

**Affiliations:** ^1^Department of Psychiatry, University of WisconsinMadison, WI, USA; ^2^Medical Scientist Training Program and Neuroscience Training Program, University of WisconsinMadison, WI, USA; ^3^Department of Psychology, University of WisconsinMadison, WI, USA

**Keywords:** consciousness, sleep, dreaming, wakefulness, EEG

## Abstract

Dreaming—a particular form of consciousness that occurs during sleep—undergoes major changes in the course of the night. We aimed to outline state-dependent features of consciousness using a paradigm with multiple serial awakenings/questionings that allowed for within as well as between subject comparisons. Seven healthy participants who spent 44 experimental study nights in the laboratory were awakened by a computerized sound at 15–30 min intervals, regardless of sleep stage, and questioned for the presence or absence of sleep consciousness. Recall without content (“I was experiencing something but do not remember what”) was considered separately. Subjects had to indicate the content of the most recent conscious experience prior to the alarm sound and to estimate its duration and richness. We also assessed the degree of thinking and perceiving, self- and environment-relatedness and reflective consciousness of the experiences. Of the 778 questionings, 5% were performed during wakefulness, 2% in stage N1, 42% in N2, 33% in N3, and 17% in rapid eye movement (REM) sleep. Recall with content was reported in 34% of non-REM and in 77% of REM sleep awakenings. Sleep fragmentation inherent to the study design appeared to only minimally affect the recall of conscious experiences. Each stage displayed a unique combination of characteristic features of sleep consciousness. In conclusion, our serial awakening paradigm allowed us to collect a large and representative sample of conscious experiences across states of being. It represents a time-efficient method for the study of sleep consciousness that may prove particularly advantageous when combined with techniques such as functional MRI and high-density EEG.

## Introduction

Dreaming is a valuable model for the study of consciousness (Kahn and Gover, 2010; Nir and Tononi, 2010). Not only is it a common and recurrent cognitive phenomenon, it also undergoes major quantitative and qualitative changes in the course of the night. Sleep consciousness can take the form of short visual hallucinations at sleep onset, often fades away during slow wave sleep at the beginning of the night and may reemerge in vivid, story-like dreams typical of REM sleep in the early morning.

Assessing conscious experiences during sleep is a challenging task. Mental activity is by definition subjective, and therefore not directly accessible to the investigator, who has to rely on retrospective reports that are obtained after awakening the subject. Most classic laboratory studies investigating conscious experiences during sleep have used paradigms with few awakenings, almost never exceeding six per night, often systematically scheduled after a fixed time in a particular sleep stage (Dement and Kleitman, 1957; Foulkes, 1962; Goodenough et al., 1965; Ogilvie et al., 1982; Foulkes and Schmidt, 1983; Williamson et al., 1986; Cicogna et al., 1991; Antrobus et al., 1995; Casagrande et al., 1996; Hobson et al., 2000). In these studies, participants were typically asked to report the whole dream or “everything that was going through their mind” prior to the awakening and additionally to answer a series of questions relating to the content of their experiences. Although this method has obvious advantages, including minimal disruption of sleep structure and optimal comparability of reports, it is also expensive and time-consuming, as the assessment has to be repeated for multiple nights or subjects in order to obtain a sufficiently large number of awakenings to allow for statistical comparisons. Also, limiting the analysis to awakenings obtained after a fixed time into a sleep stage may compromise the representativeness of the sample of conscious experiences. More recently, a few studies with frequent serial awakenings have been published. However, these were either limited to the falling asleep period (Horikawa et al., 2013), the first non-REM (NREM) sleep cycle of the night (Noreika et al., 2009), or distributed over a 24-h time frame (Chellappa et al., 2011). Only a few groups compared conscious experiences during wakefulness and sleep in the same individuals (Kahan et al., 1997; Fosse et al., 2001; Stickgold et al., 2001; Kahan and Laberge, 2011).

The aim of the current study was to validate a study paradigm with multiple serial questionings performed irrespective of sleep stage, at pseudorandom intervals, and also during wakefulness. In order to be more time efficient and to increase the probability that the experience occurred immediately before the questioning, only the “most recent conscious experience” before the alarm sound was assessed. In an attempt to dissociate consciousness from recall (Strauch and Meier, 1996), conscious experiences with and without recall of content were considered separately. This serial awakening paradigm has several advantages. First, obtaining a high number of samples per individual allows for *within* subject comparisons of conscious experiences. In particular, it becomes possible to define not only the characteristics that are common to all dreams and individuals, but also what is specific to a particular dream and person (Nir and Tononi, 2010). Second, performing awakenings irrespective of stage and of the time spent in a sleep stage allows researchers to maximally exploit the variety of conscious states that is inherent to sleep. Finally, this paradigm may prove particularly useful for studies using expensive techniques with a complex setup, such as functional MRI (fMRI) and high-density EEG (hdEEG), which can benefit from minimizing the number of study nights.

## Methods

### Subjects

We included seven healthy subjects [3 males, age 31 ± 8.8 years, 21–47 (mean ± *SD*, range)] who were screened for neurological, psychiatric and sleep disorders during a structured medical interview. None of the subjects was on psychotropic medication. All the participants had a good sleep quality as assessed by the Pittsburgh Sleep Quality Index (<5 points) and scored within normal limits on the Epworth Sleepiness Scale. A personal interest in dreaming or frequent recall of dreams was not a prerequisite. The present study was conducted as part of a larger research project that was approved by our Institutional Review Board. Written informed consent was obtained from each participant.

### Procedure

Two weeks prior to the overnight recordings, subjects received detailed instructions regarding the questionnaire used in the study. They were asked to fill it out in the morning upon awakening at home for this period, in order to become familiar with reporting mental activity. Six overnight recordings in the laboratory were scheduled for each participant, with a maximum of three consecutive nights (consecutive nights are referred to as one session throughout the text) and a minimal interval of 1 week between sessions. Recordings were started between 10 pm and 12 am, depending on the participant's habitual sleep schedule, and wake time was set between 6 and 8 am. Questionings were carried out pseudorandomly every 15–30 min, irrespective of sleep stage and of whether the subject was asleep or awake. Participants were alerted by a computerized sound lasting 1.5 s. They were instructed to signal that they had heard the sound and to then lie quietly on their back with eyes closed. Interviews were conducted via intercom using a structured questionnaire, and answers were audiotaped and later transcribed. To limit the effects of sleep restriction resulting from the study design, after each study night participants were allowed to go back to sleep unrecorded until 12 pm.

In order to determine how the frequency of the awakenings may have affected the recall of conscious experiences, one subject underwent four additional nights in which the interval between awakenings was lengthened to 30–45 min.

### Questionnaire

The interview based on the questionnaire lasted between 20 s and 3.5 min, depending on whether the subject reported a conscious experience and had to answer additional questions related to the content. The features that were assessed by means of the questionnaire are described below.

#### Presence or absence of conscious experiences

The first question participants were asked was: “What was the last thing going through your mind prior to the alarm sound?” (i.e. “What was the last thing going through your mind prior to the alarm sound?”). Because of time constraints inherent to the study design, subjects were instructed to report only the *most recent* conscious experience (i.e. image, thought or scene) they had before the alarm sound instead of the *whole* experience. Experience was defined as “any kind of mental activity,” which included thoughts, dreams, perceptions, emotions, etc. Three possible answers were considered: (1) no conscious experiences (NCE), (2) conscious experiences without recall of content (CEWR), when the subject had experienced something but could not remember the content, and (3) conscious experiences (CE), when the participant could describe the content of the experiences. In case of NCE or CEWR, the interview ended and the subject was told to go back to sleep.

#### Quantitative features of CE

In an attempt to quantify CE, we asked participants to estimate the length and complexity of the experience by means of four questions. For all four of these questions, subjects had been instructed to provide answers in units of time (i.e., seconds, minutes, or hours).

1. Duration of CE: Participants were asked: *“For how long were you having continuous experiences before the alarm sound?”* They were instructed to give an estimation of how long they were experiencing something before the alarm sound. It was pointed out to subjects that they did not need to remember the exact content of the experiences and also that this question did not necessarily refer to the time that they thought had elapsed since the last questioning.
2. Duration of most recent CE: The question *“How long did the most recent experience last?”* referred to the most recent experience before the alarm sound, the content of which was assessed by the first question.
3. Recall back in time: With the question *“How far back in time can you specifically recall?”* we aimed at assessing the “narrative thread” and “continuity” of the conscious experience. Subjects had to give an estimate of how far back in time they could recall the content of the experience.
4. Richness and complexity of CE: Subjects were asked: *“How rich and complex was the experience? How long would it take to recount it?”* They were told that this estimate was different from the duration of the experience. A dream about driving on a boring road for hours, for instance, with nothing else happening, would be a dream with a long duration but low richness, which could probably be recounted in 5 s.

#### Cognitive dimensions of CE

We assessed five cognitive dimensions, which were chosen, based on the literature, because they were shown to demonstrate state-dependent variability and regionally specific patterns of brain activity.

Participants were instructed to evaluate the degree of *thinking and perceiving* related to the conscious experience on a 5-point scale ranging from 1 (none) to 5 (maximal). This scale was adapted from a recent study on CE during wakefulness (Vanhaudenhuyse et al., 2011). We phrased the questions in the following way: (1) “How much on a scale from 1 to 5 were you perceiving rather than thinking?” and (2) “How much on a scale from 1 to 5 were you thinking rather than perceiving?” The thinking score was then subtracted from the perceiving score to obtain a composite thinking/perceiving score ranging from −4 (maximal thinking, minimal perceiving) to +4 (maximal perceiving, minimal thinking).

The degree to which CE related to *self and environment* was assessed by asking: (1) “To what degree was this experience centered on yourself rather than on the environment?” and (2) “To what degree was this experience centered on the environment rather than on yourself?” Again, answers were given on a 5-point scale, ranging from 1 (none) to 5 (maximal). A composite self/environment score was obtained by subtracting the self-score from the environment score, so that possible values ranged from −4 (maximal self-relatedness, minimal environment-relatedness) to +4 (maximal environment-relatedness, minimal self-relatedness). It was pointed out to subjects that these questions were related but not identical to the questions concerning thinking and perceiving. For example, one might strongly perceive something related to the self rather than to the environment, like pain coming from a bleeding wound on one's hand, without thinking about it (high perceiving, high self relatedness); conversely, one might think about something completely unrelated to the self without perceiving it, as would be the case when one mentally performs mathematical operations (high thinking, low self-relatedness).

*Reflective consciousness* was assessed using a scale adapted from a previous study (Fosse, 2000). Participants were asked: (1) “To what degree were you aware that these experiences were not real?” and (2) “To what degree did you have voluntary control over the content of the experience?” Answers were given on a scale ranging from 1 (not at all) to 5 (fully). Both scores were added to obtain a composite score of reflective consciousness, ranging from 2 (minimal reflective consciousness) to 10 (maximal reflective consciousness).

#### Other questions

Other items in the questionnaire, which will not be further discussed in the present article, referred to the presence of specific categories of content and to the subjective estimation of the state of being (asleep or awake).

### Recordings

Recordings were performed using hd-EEG with a 256-channel system (Electrical Geodesics), electro-oculography (four of the 256 electrodes placed at the outer canthi of the eyes were used to monitor eye movements) and submental electromyography. The sampling frequency was 500 Hz. Participants were continuously videotaped during the sleep period. Sleep scoring was performed over 30 s-epochs according to standard criteria (Iber et al., 2007).

### Statistical analysis

Statistical analyses were performed using STATISTICA 8.0 (StatSoft^©^).

To determine whether CE and NCE differed significantly with respect to time of the night and time spent within a sleep stage, we first calculated the mean of the variables “time since lights out” and “time spent in stage” for each category (CE and NCE). This was done for each subject and for REM and stages N2/N3 separately. We then compared CE and NCE using paired *t*-tests.

To determine whether in one subject, the proportion of CE, CEWR and NCE differed between nights with frequent awakenings and nights with less frequent awakenings, we used a Chi-square test of independence for stages N2/N3 and REM and for each subject separately.

To compare the proportion of CE, CEWR and NCE between first nights and subsequent nights in a session we also used a Chi-square test of independence, again for stages N2/N3 and REM and for each subject separately.

To evaluate the effect of stage (wakefulness, N1, N2/N3, REM) on characteristics of CE (duration richness, reflective consciousness, thinking/perceiving, self/environment-relatedness), we used a mixed effect model (as a multivariate analysis), with two independent variables (subject and stage), and 7 dependent variables (dream characteristics). Stage was defined as a fixed factor and subject as a random factor (i.e. as a source of random variability, Keppel and Wickens, 2004). Follow-up paired *t*-tests were conducted on the mean values of the dream characteristics for each subject and stage to evaluate pairwise differences between stages. None of the variables considered deviated significantly from the normal distribution as indicated by Kolomogoroff–Smirnoff tests. A *p* < 0.05 was considered significant for all the analyses. Note that all the analyses were carried out for N2 and N3 separately. However, because considering these stages separately or together did not significantly change the results, we reported results for N2/N3 together, in order to simplify their presentation.

## Results

In the present article, we will focus on the phenomenology of CE across different states of being. The results of the EEG analyses will be the subject of a separate article.

Of the seven subjects, five completed the whole six-night protocol. One subject had to postpone the sixth night because of personal obligations, while the other participant felt uncomfortable wearing the net and for this reason terminated two of the nights prematurely and cancelled the sixth night. One subject presented two spontaneous confusional arousals out of slow wave sleep during the second night of a session.

### Questionings

On the whole, 785 questionings were performed during 44 study nights. Two questionings out of slow wave sleep were excluded from the analysis because subjects were too somnolent or confused to understand and answer any of the questions. Five other questionings were excluded because of technical problems with the amplifier. A subset of 37 questionings (four study nights) will be considered separately because they were performed with longer time intervals between questionings.

**Figure 1:**
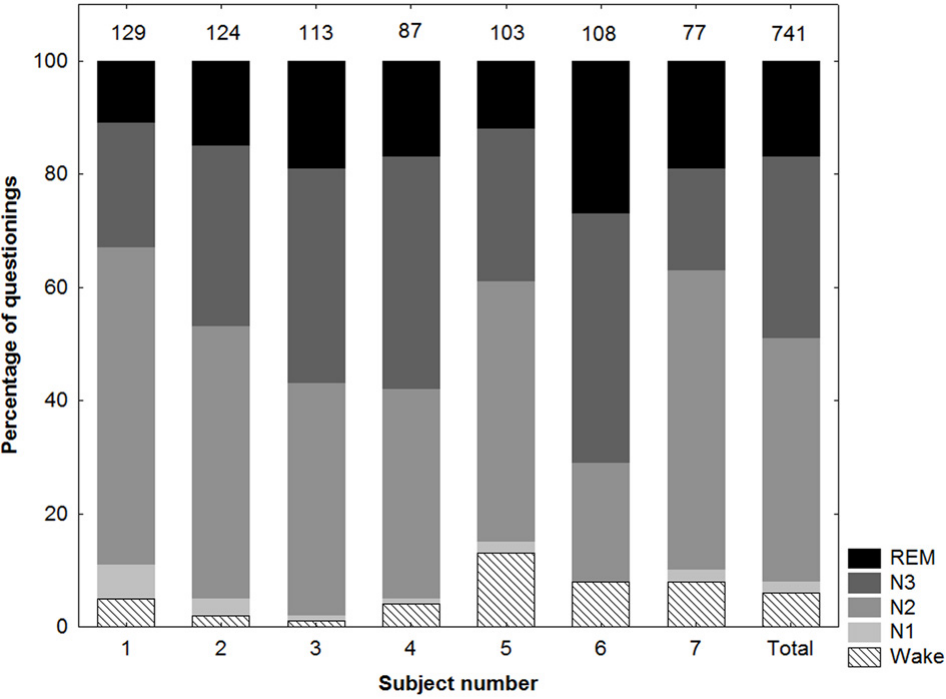
**Distribution of questionings among subjects and stages.** The number at the top of each bar indicates the total number of questionings per subject.

**Figure 2:**
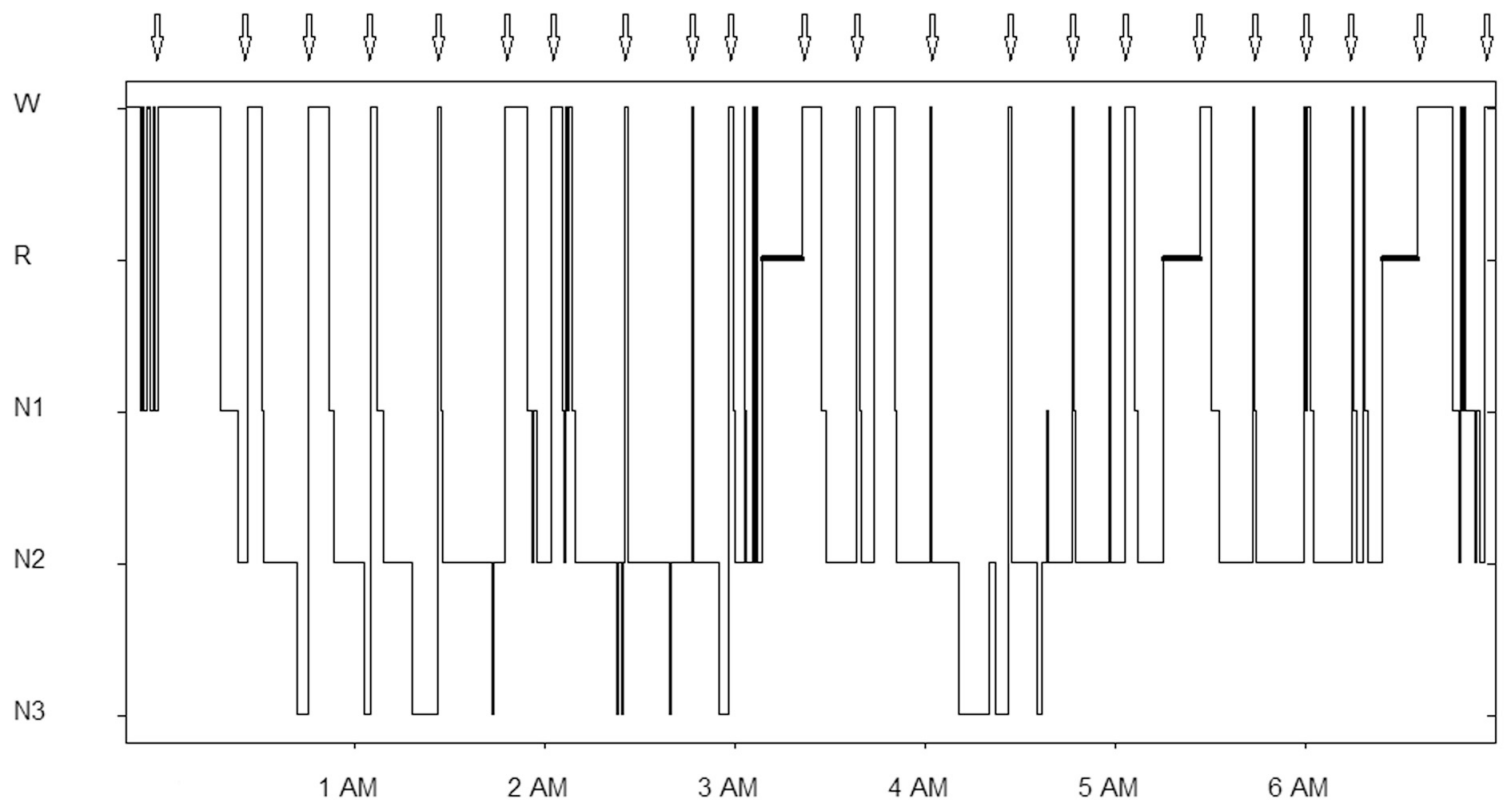
**Hypnogram of a study night with 22 questionings (indicated by arrows at the top of the graph).** W, wakefulness; R, REM sleep, N1, stage N1; N2 stage N2; N3, stage N3.

The distribution of the remaining 741 questionings among subjects and stages is shown in Figure 1. Mean interval between questionings was 23.1 min (±8.3), mean number of questionings per night 18.2 (±3.4). A summary of polysomnographic parameters is presented in Table 1. A typical hypnogram of a study night is shown in Figure 2.

**Table 1:** **Mean and standard deviation (SD) of polysomnographic parameters (seven subjects, 44 study nights)**.

**Sleep parameter**	**Mean (±***SD***)**
Total sleep time (min)	305 ± 37
Sleep efficiency (%)	73 ± 8
WASO (min)	110 ± 36
N1 (min)	26 ± 9
N1 (%)	10 ± 6
N2 (min)	174 ± 47
N2 (%)	59 ± 12
N3 (min)	64 ± 36
N3 (%)	21 ± 12
REM (min)	33 ± 12
REM (%)	11 ± 4

*Sleep efficiency = 100 × (total sleep time/sleep period time). WASO, wake after sleep onset. The proportion of stages is expressed as the percentage of total sleep time*.

### Presence and absence of CE

**Figure 3:**
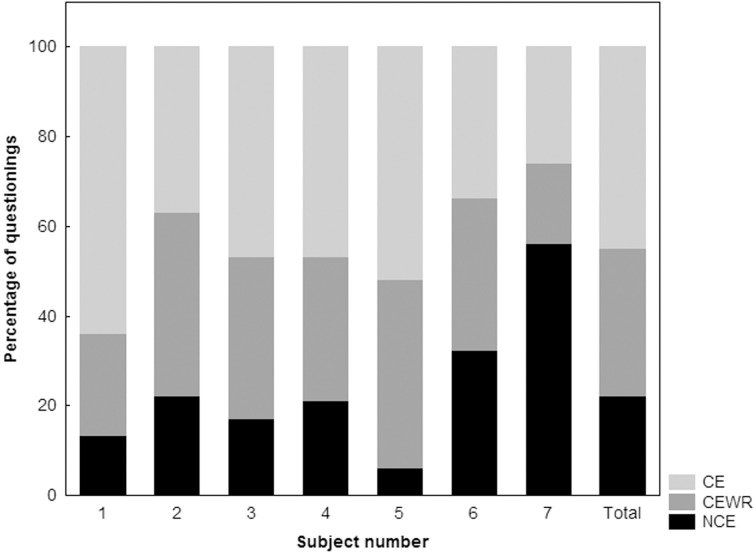
**Proportion of conscious experiences (CE), conscious experiences without recall (CEWR) and no conscious experiences (NCE) across subjects**.

**Figure 4:**
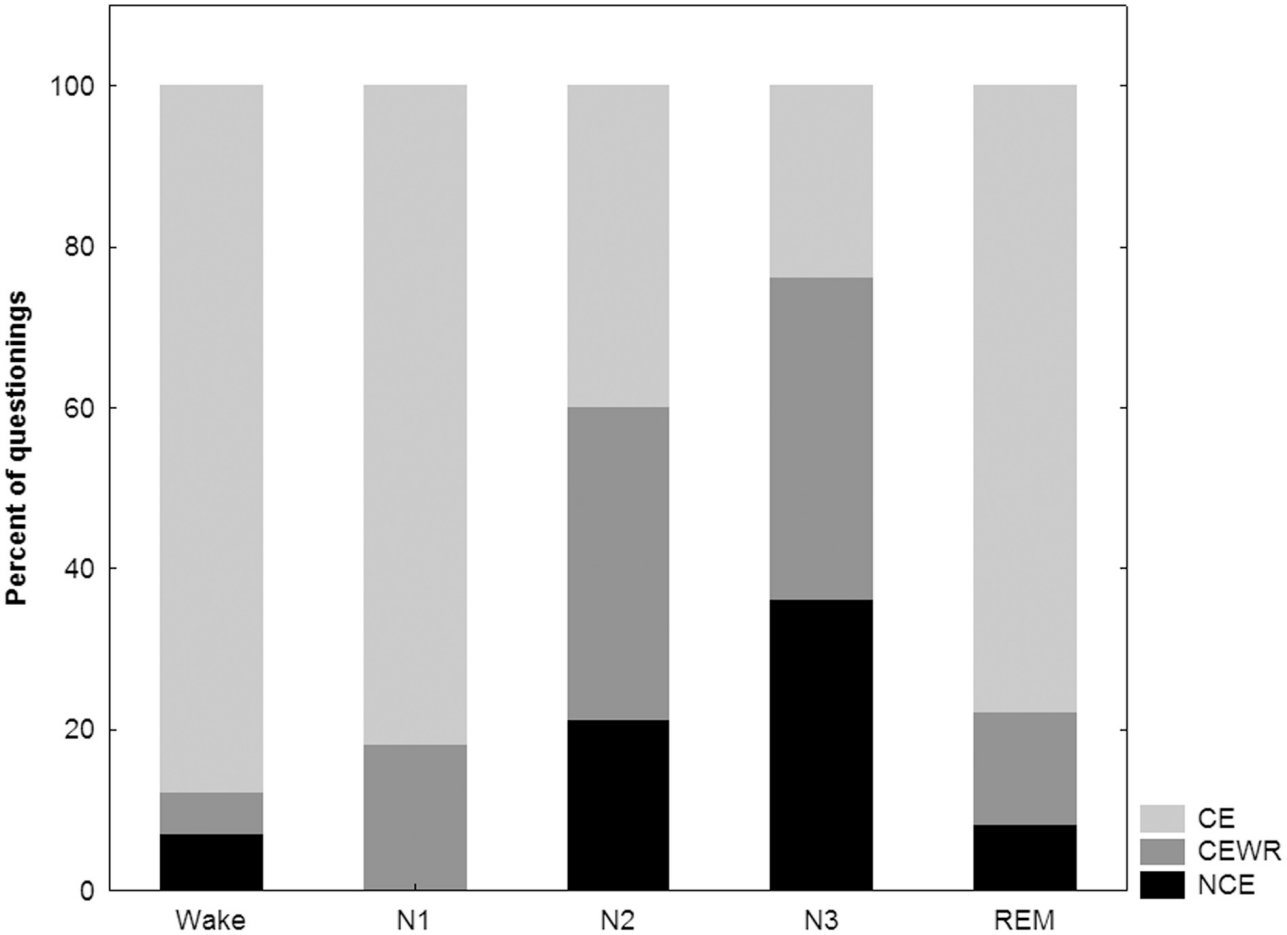
**Proportion of conscious experiences (CE), conscious experiences without recall (CEWR) and no conscious experiences (NCE) across stages**.

Of the 741 questionings, 334 (45%) were associated with CE, 242 (33%) with CEWR, and 164 (22%) with NCE. Recall of CE with content was 96 ± 7% (83–100) for wakefulness, 77 ± 39% (0–100) for N1, 42 ± 15% (22–64) for N2, 23 ± 15% for N3 (0–48) and 82 ± 19% for REM (41–100). How the proportions of CE, CEWR and NCE differed between subjects and stages is shown in Figures 3 and 4 respectively.

#### Effect of time of the night and time spent in stage

**Figure 5:**
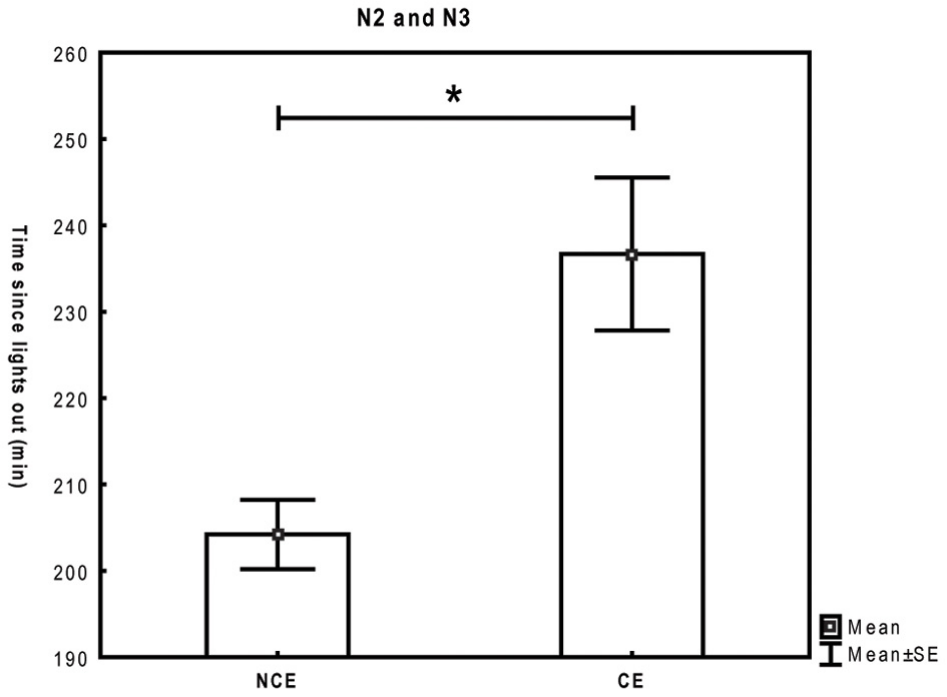
**Time since lights out (mean and standard error of mean) for questionings associated with conscious experiences (CE) and no conscious experiences (NCE) in stages 2 and 3 (seven subjects).** Paired *t*-test. ^*^p < 0.05.

**Figure 6:**
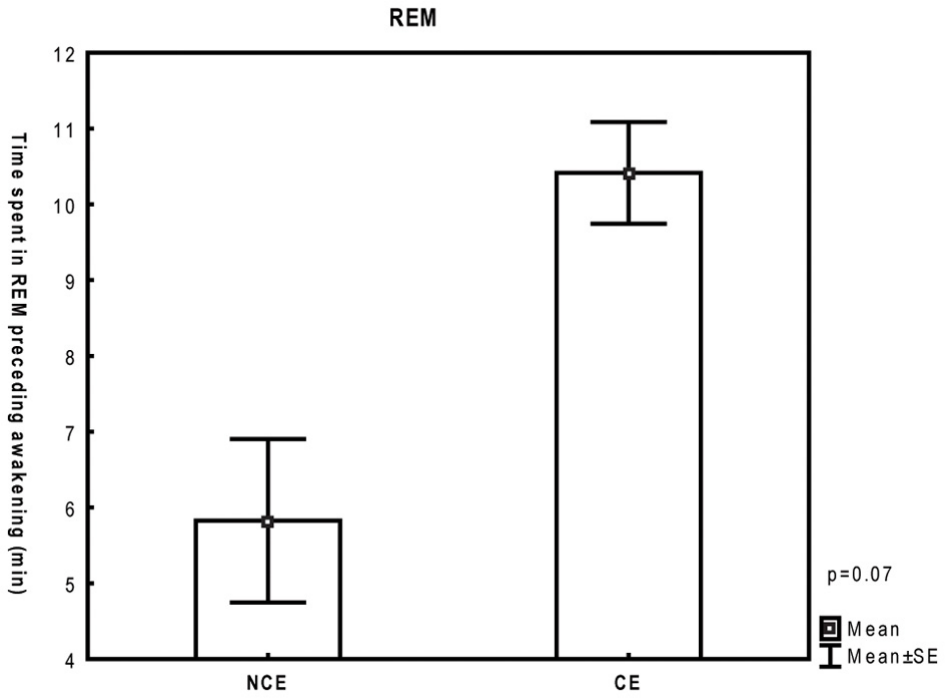
**Time spent in REM sleep (mean and standard error of mean) for questionings associated with conscious experiences (CE) and no conscious experiences (NCE) in REM sleep (four subjects).** Paired *t*-test. ^*^p = 0.07.

In N2/N3, CE and NCE significantly differed with respect to the time of the night, with CE occurring later than NCE [*t*_(6)_ = −2.9, *p* = 0.03] (Figure 5), while there was no difference with respect to time spent in stage [*t*_(6)_= 1.34, *p* = 0.2]. Only four of the subjects had both CE and NCE in REM sleep and could thus be included in the analysis. Results indicated a trend for CE to occur after a longer time spent in REM sleep than NCE [*t*_(3)_ = −2.7, *p* = 0.07], (Figure 6). CE and NCE in REM sleep did not differ significantly with respect to time of the night [*t*_(3)_ = −1.6, *p* = 0.2].

#### Effect of frequency of awakenings

To determine whether the frequency of awakenings had an effect on the proportion of CE, we compared two different conditions in one subject: six nights with frequent awakenings (129 questionings, 21.5 ± 1.76 questionings per night, interval between questionings 19.5 ± 4.3 min) and four nights with less frequent awakenings (37 questionings, 9.3 ± 2.2 questionings per night, interval between questioning 35.3 ± 13.8 min). The proportions of CE, NCE and CWR in a given sleep stage did not differ significantly in the two conditions [*X*^2^_(1)_ = 2.39, *p* = 0.3 and *X*^2^_(1)_ = 0.74, *p* = 0.7, for N2/N3 and REM sleep respectively].

#### Effect of first vs. subsequent nights

To assess how the sleep restriction inherent to our study design affected the recall of CE, we compared the proportion of CE in the first study night of a session (with presumably minimal sleep restriction) to subsequent nights in the same session (with presumably greater sleep restriction resulting from sleep fragmentation during the previous nights). We found that in two of the seven subjects, the proportion of CE in N2/N3 was significantly higher in the first study night compared to subsequent nights of the same session. In one subject, the proportion of CE and CEWR decreased from 38 to 22% and from 62 to 48% respectively, while NCE increased from 0 to 30% [*X*^2^_(1)_ = 8.4, *p* = 0.01]. In the other subject, the relative amount of CE and CEWR decreased from 19 to 0% and from 24 to 14% respectively, while the relative amount of NCE increased from 0 to 30% [(*X*^2^_(1)_ = 6.7, *p* = 0.03].

#### Learning effect

Only one of the seven subjects presented a significant increase of the proportion of CE in the course of the experiment, which was limited to stage N2 [*X*^2^_(7)_ = 17.885, *p* = 0.02], suggesting that overall, there was no major learning effect on the recall of CE.

### Characteristics of CE

A significant main effect of stage (i.e. a significant difference in means between stages), but not of subject was observed for recall back in time [*F*_(3)_ = 3.5, *p* = 0.04], richness of CE [*F*_(3)_ = 3.8, *p* = 0.03], the thinking/perceiving dimension [*F*_(3)_ = 9.2, *p* < 0.001] and reflective consciousness [*F*_(3)_ = 29.3, *p* < 0.001]. For the duration of CE and the self/environment dimension, the effect of stage was only marginally significant ([*F*_(3)_ = 2.8, *p* = 0.07] and [*F*_(3)_ = 2.7, *p* = 0.08] respectively). No main effect of stage was found for the duration of the last CE [*F*_(3)_ = 0.7, *p* = 0.6].

A significant effect of subject (i.e. a significant variability between subjects) was found for duration of CE [*F*_(6)_ = 6.6, *p* < 0.01] and for the duration of the last conscious experience [*F*_(6)_ = 6.0, *p* < 0.01]. A significant interaction between subject and stage was found for all the variables [*F*_(15)_ > 2.2, *p* < 0.05], meaning that the variability among subjects varied significantly between stages in all cases. This effect is not of primary interest in the present study, but is reported for the sake of completeness.

**Figure 7:**
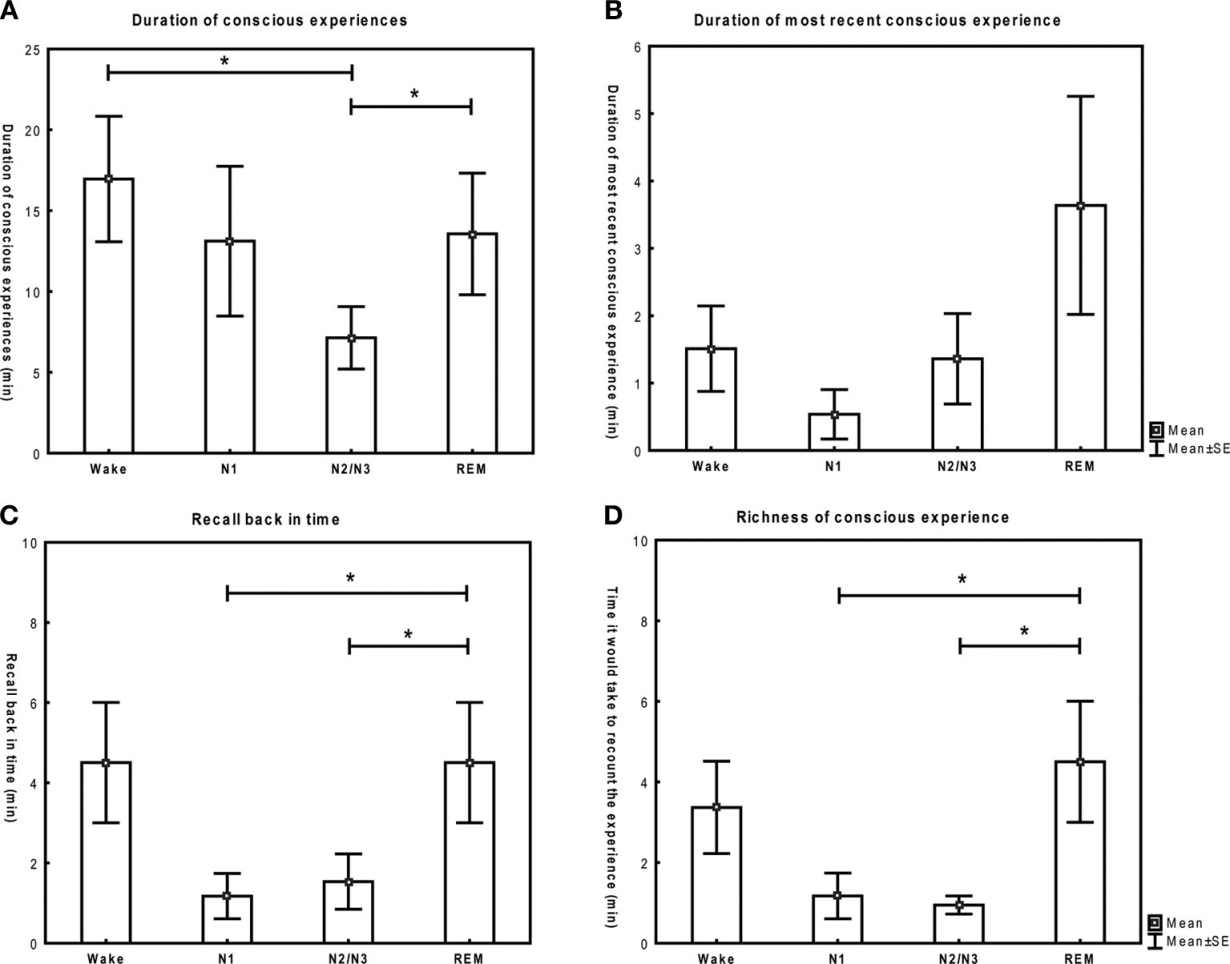
**(A–D)** Quantitative features of conscious experiences across stages (mean and standard error). Paired *t*-tests. ^*^p < 0.05. Wake: 6 subjects, N1: 5 subjects, N2/N3: 7 subjects, REM: 7 subjects.

**Figure 8:**
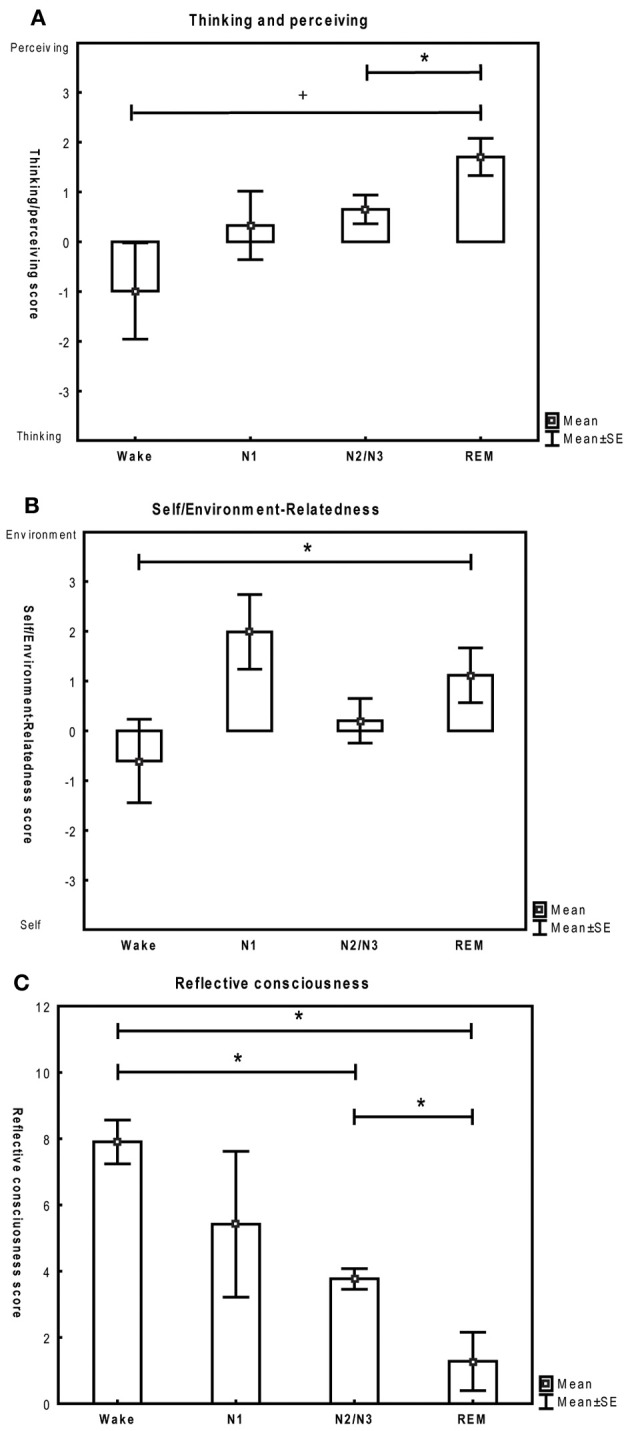
**(A–C)** Qualitative features of conscious experiences across stages (mean and standard error). Paired *t*-tests. ^*^p < 0.05, ^+^ *p* = 0.05. Wake: 6 subjects, N1: 5 subjects, N2/N3: 7 subjects, REM: 7 subjects.

How characteristics of CE differed among stages and results of post-hoc analyses are presented in Figures 7 and 8. Table 2 provides a conceptual summary of the results. Representative examples of reports and their distinguishing features are displayed in Table 3.

**Table 2:** **Summary of characteristic features of conscious experiences across stages**.

	**Duration of CE**	**Recall back in time**	**Richness**	**Thinking/Perceiving**	**Self/Environment-relatedness**	**Reflective consciousness**
Wakefulness	High	High	Intermediate	Thinking	Self	High
N1	High	Low	Low	Intermediate	Environment	Intermediate
N2/3	Low	Low	Low	Intermediate	Intermediate	Intermediate
REM	High	High	High	Perceiving	Environment	Low

**Table 3:** **Illustrative examples of most recent conscious experiences and their characteristics**.

**Most recent CE**	**Time**	**Sleep stage**	**Min in stage**	**Duration of CE**	**Recall in time**	**Richness**	**Thinking/perceiving**	**Self/environment-relatedness**	**Reflective consciousness**
I was thinking about a restaurant I went to yesterday.	3:34 am	Wake	33	20 min	2 min	20 s	−4	0	10
I was in a living room, with lots of flowers and insects.	11:44 pm	N1	2	30 min	3 min	15 s	2	3	7
I saw a facebook notification saying that a friend from high school was engaged.	4:33 am	N2	4	30 s	20 s	30 s	2	2	2
I was eating bacon.	5:44 am	N3	6	1 min	1 min	30 s	2	−1	3
I was looking at a giant vulture. It was looking at us through the window, in a very mean way.	3:26 am	REM	11	20 min	20 min	15 min	2	2	2

*The thinking/perceiving score ranges from −4 (maximal thinking, minimal perceiving) to +4 (maximal perceiving, minimal thinking). The reflective consciousness score ranges from 2 (minimal reflective consciousness) to 10 (maximal reflective consciousness). The self/environment score ranges from −4 (maximal self-relatedness, minimal environment-relatedness) to +4 (maximal environment-relatedness, minimal self-relatedness)*.

## Discussion

In the present study we investigated state-specific features of CE using a serial awakenings method in which questionings were performed irrespective of sleep stage and also during wakefulness. Despite the frequent awakenings, subjects could fall back asleep repeatedly and rapidly, allowing us to perform awakenings in every sleep stage. In fact, aside from the relatively high proportion of awakenings in N3, the distribution of awakenings among sleep stages (Figure 1) roughly parallels the typical distribution of sleep stages during a night of normal sleep (Ohayon et al., 2004).

Overall, the results we obtained with this paradigm are in good agreement with the literature. In particular, the proportion of questionings yielding reports of CE is comparable to results of previous studies. Reports of CE in our study were obtained in 96 ± 7% of questionings during wakefulness [98% in a previous study (Foulkes and Fleisher, 1975)], in 77 ± 39 % in N1 [85–98% in the literature (Foulkes, 1962; Foulkes and Vogel, 1965; Rowley et al., 1998; Stickgold et al., 2001)], in 42 ± 15 % in N2 and in 23 ± 16% in N3 [42 ± 21% in N2/N3 in an review of 33 NREM studies (Nielsen, 1999)], and in 82 ± 19% in REM sleep [82 ± 9% in a review of 29 REM studies (Nielsen, 1999)]. Also, the characteristics of CE across stages are consistent with previous work. The word count of reports of CE is typically highest in REM sleep, followed by wakefulness, stages N2/N3 and finally sleep onset (Stickgold et al., 2001). In the present study, the variables “richness” and “recall back in time” show this same pattern across stages. This is not surprising, considering that these quantitative variables relate specifically to the whole content of the dream, just like the total word count. The other quantitative variable “duration of CE” shows a different distribution: although highly variable between subjects, it tends to be highest in wakefulness, followed by stage 1 and REM sleep and shortest in N2/N3. The dissociation between a long “duration of CE” and lower “richness” and “recall back in time” at sleep onset and to a lesser extent in wakefulness suggests that consciousness on the whole remains present for an extended period of time in these stages but that individual CE relating to a particular context are short and disconnected from each other. Indeed, it is well known that hypnagogic images at sleep onset are short and resemble a series of “snapshots” (Nir and Tononi, 2010) and that mental activity during wakefulness appears to contain more abrupt topic changes when compared to REM sleep (Reinsel et al., 1992). We did not find a main effect of stage on the duration of the “most recent conscious experience.” The fact that subjects reported similar durations of the “last experience,” for each stage suggests that subjects were consistent throughout the experiment with what they considered to be the “last experience.”

We found that thinking was highest during wakefulness and that perceiving was elevated in REM sleep and to a lesser extent in N2/N3, while sleep onset experiences were characterized by intermediate scores for both dimensions. These results are consistent with studies that have assessed the thought-like vs. hallucinatory character of mental activity across sleep stages (Foulkes, 1962; Goodenough et al., 1965; Rowley et al., 1998; Fosse et al., 2001, 2004). Similar to previous studies, we found that reflective consciousness decreased from wakefulness through progressively deeper sleep stages (Foulkes and Vogel, 1965), reaching its lowest value in REM sleep. As for the self/environment dimension, only a marginally significant main effect of stage was found. Self-related experiences tended to be more prominent during wakefulness, while environment-related mental activity prevailed during REM sleep and N1. To the best of our knowledge, these characteristics have not been previously assessed by a single score comprising both dimensions. However, thoughts about one's own behavior have been shown to be prominent during wakefulness (Kahan and Laberge, 2011), while the dreamer is known to have a passive, observer-like quality in hypnagogic experiences (Schacter, 1976). Although the self is said to be present in 90% of REM reports, only 3% of the semantic content of the dream describes the dream self, the rest being related to objects, actions, person or places (Revonsuo and Salmivalli, 1995), suggesting that the environment dimension is more prominent when directly compared to the self dimension in REM reports.

It is likely that the sleep fragmentation and restriction inherent to our study paradigm resulted in increased intensity of sleep inertia upon awakening and that this in turn influenced the recall of CE. In two subjects for instance, we found that the recall rate in N2/N3 was reduced during the second and third nights of a session, compared to the first night, when there were presumably minimal effects of sleep restriction. Also, two questionings had to be excluded because subjects were too somnolent to understand and answer any of the questions, and one participant presented two spontaneous confusional arousals out of slow wave sleep. However this effect seems to be limited, as it was observed only for a small minority of awakenings. Also, the proportion of CE in N2/N3 was highest late in the night, suggesting that the increasing sleep fragmentation did not have a major influence on recall. Additionally, nights with frequent and less frequent awakenings did not differ significantly with regard to recall of CE. Finally, the fact that our observations are largely consistent with the literature suggests that this paradigm yields valid results. This method may be particularly advantageous for studies using complex and expensive techniques (i.e. hdEEG, fMRI), in which minimizing the number of study nights represents a substantial advantage. Nevertheless, the sleep fragmentation can be uncomfortable and one subject prematurely ended the protocol for this reason. The serial awakening paradigm is thus best reserved for highly motivated individuals with a good sleep quality.

## Conclusion

In conclusion, our paradigm allowed us to obtain multiple reports of several individuals in all stages and to outline state-specific features of consciousness. The sleep restriction inherent to this method appears to have only minimally affected recall of CE. Our results suggest that this method provides a valuable tool for the study of sleep consciousness, especially when minimizing the number of study nights is a priority.

### Conflict of interest statement

The authors declare that the research was conducted in the absence of any commercial or financial relationships that could be construed as a potential conflict of interest.
